# Influence of the design of 3D-printed indirect bonding trays and experience of the clinician on the accuracy of bracket placement

**DOI:** 10.1007/s00056-024-00517-2

**Published:** 2024-02-27

**Authors:** Hisham Sabbagh, Lea Hoffmann, Andrea Wichelhaus, Andreas Kessler

**Affiliations:** 1 Department of Orthodontics and Dentofacial Orthopedics, LMU University Hospital, LMU Munich, Goethestraße 70, 80336 Munich, Germany; 2 Department of Conservative Dentistry and Periodontology, LMU University Hospital, LMU Munich, Goethestraße 70, 80336 Munich, Germany

**Keywords:** Learning curve, Tray design, Bracket placement accuracy, 3D printing, Orthodontic brackets, Lernkurve, Tray-Design, Genauigkeit bei der Bracketplatzierung, 3‑D-Druck, Kieferorthopädische Brackets

## Abstract

**Purpose:**

The aim was to investigate the influence of three different three-dimensional (3D)-printed bonding tray designs and professional experience on accuracy of indirect bracket placement.

**Methods:**

Virtual bracket placement was performed on a scanned dental model using OnyxCeph software (Image Instruments, Chemnitz, Germany). Three different designs for indirect bonding trays (open, semi-open, and closed design) were created and produced using a 3D printer. To analyze the influence of professional experience, one of the three tray designs was produced twice. In this case, bracket placement was performed by an inexperienced dentist. Bracket positions were scanned after the indirect bonding procedure. Linear and angular transfer errors were measured. Significant differences between the target and actual situation were analyzed using the Kruskal–Wallis and χ^2^ test.

**Results:**

All bonding tray designs resulted in comparable results. The results of the unexperienced dentist showed significantly higher deviations than those for the experienced orthodontist in the torque direction. However, the mean values were comparable. The open tray design exceeded the clinically acceptable limits of 0.25 mm and 1° more often. The inexperienced dentist exceeded these limits significantly more often than the experienced orthodontist in the vertical and torque direction. The immediate bracket loss rate showed no significant differences between the different tray designs. Significantly more bracket losses were observed for the inexperienced dentist during the procedure compared to the experienced orthodontist.

**Conclusions:**

The bonding tray design and professional experience had an influence on the exceedance of clinically relevant limits of positioning accuracy and on the bracket loss rate.

## Zuammenfassung

**Ziel:**

Untersuchung des Einflusses von 3 verschiedenen Designs 3‑D-gedruckter Brackettransferschienen und der Berufserfahrung auf die Genauigkeit der indirekten Bracketplatzierung.

**Methoden:**

Die virtuelle Bracketplatzierung wurde mit der OnyxCeph-Software (Image Instruments, Chemnitz, Deutschland) auf einem gescannten Zahnmodell durchgeführt. Drei verschiedene Designs für indirekte Brackettransferschienen (offenes, halboffenes und geschlossenes Design) wurden entworfen und mit einem 3‑D-Drucker hergestellt. Um den Einfluss der Berufserfahrung zu analysieren, wurde eines der 3 Designs 2‑mal hergestellt. In diesem Fall wurde die Bracketplatzierung von einem kieferorthopädisch unerfahrenen Zahnarzt durchgeführt. Die Bracketpositionen wurden nach dem indirekten Klebeverfahren gescannt. Es wurden lineare und angulare Übertragungsfehler gemessen. Signifikante Unterschiede zwischen Soll- und Ist-Situation wurden mithilfe des Kruskal-Wallis- und des χ^2^-Tests analysiert.

**Ergebnisse:**

Alle Designs führten zu vergleichbaren Ergebnissen. Die Ergebnisse des kieferorthopädisch unerfahrenen Zahnarztes zeigten signifikant höhere Abweichungen bei der Bracketinklination. Die Mittelwerte waren jedoch vergleichbar. Das offene Tray-Design überschritt die klinisch akzeptablen Grenzen von 0,25 mm und 1° häufiger. Der kieferorthopädisch unerfahrene Zahnarzt überschritt diese Grenzwerte signifikant häufiger als der erfahrene Kieferorthopäde in vertikaler Richtung und hinsichtlich der Bracketinklination. Bei der Rate der sofortigen Bracketverluste zeigten sich keine signifikanten Unterschiede zwischen den verschiedenen Tray-Designs. Bei dem kieferorthopädisch unerfahrenen Zahnarzt wurden signifikant mehr Bracketverluste während der indirekten Bracketplatzierung beobachtet als bei dem erfahrenen Kieferorthopäden.

**Schlussfolgerungen:**

Das Design des Klebetabletts und die Berufserfahrung hatten Einfluss auf die Überschreitung klinisch relevanter Grenzen der Positioniergenauigkeit und auf die Bracketverlustrate.

## Introduction

In fixed orthodontic therapy, brackets, bands and buccal tubes are used to transfer forces and moments to teeth and thereby induce tooth movements. The accurate positioning of orthodontic brackets plays a crucial role, especially when using preadjusted straight wire brackets, since deviations from the correct bracket positions can lead to undesirable tooth movements, poorer treatment results, and a prolonged treatment period [3, 6]. Brackets and buccal tubes can be positioned on the teeth either directly or indirectly via a transfer aid (bonding tray) [11, 23]. Advantages of indirect bonding compared to the direct bonding technique have been described in literature, in particular higher accuracy of bracket position [1, 15, 16, 22, 25], reduced chair time, and greater patient comfort [9, 10].

Nowadays computer-aided planning and manufacturing technology enables virtual planning of bracket positions. Due to additive manufacturing (three-dimensional [3D] printing), a cost-effective and easy manufacturing of bonding trays is possible [12]. Authors of recent publications in the literature conclude that 3D printed bonding trays achieved comparable results in comparison to the gold standard polyvinyl siloxane (PVS) trays regarding bracket positioning accuracy [12, 20]. Furthermore, a systematic review on indirect bonding including 16 studies showed that the overall accuracy of the indirect bonding technique can be considered clinically acceptable [20].

However, it should be noted that this technique was also shown to involve increased time on the computer (mostly by the dentist), higher initial costs, and a higher bracket loss rate compared to the direct technique [5, 8, 13]. Furthermore, many factors can influence the accuracy of bracket transfer: choice of the brackets and their retention, the chosen 3D printer, printing direction, the 3D printing material, postprocessing, experience of the dentist etc. To date, there is limited literature on these influencing factors [20, 24].

Thus, the aim of this study was to analyze the influence of the design of the 3D printed bonding trays on the accuracy of bracket placement. Furthermore, the influence of professional experience on accuracy of bracket placement and on the immediate bracket loss rate should be analyzed.

## Materials and methods

### Virtual bracket bonding and bonding tray design

A digitized standard upper jaw model (frasaco AG‑3 WOK series, frasaco GmbH, Tettnang, Germany) was used for virtual bracket placement of orthodontic twin brackets (Mini Sprint II, Forestadent GmbH, Pforzheim, Germany) using the FA_bonding 3D module of the OnyxCeph^3^™ software (Image Instruments GmbH, Chemnitz, Germany). The brackets were virtually positioned on the centers of the clinical crowns according to Andrews using a software algorithm and subsequent manual corrections [2].

**Fig. 1:**
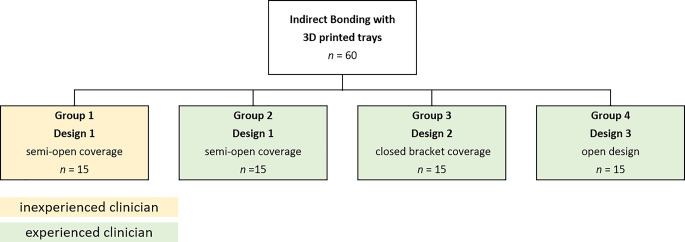
Flowchart depicting methodology and group allocation. Group 1 and group 2 share the same design (semi-open coverage) but were bonded by clinicians with differing experience. Groups 2, 3, and 4 vary in design, but were bonded by the same experienced clinician Flussdiagramm zur Darstellung von Methodik und Gruppenzuordnung. Gruppe 1 und Gruppe 2 haben das gleiche Design (halboffene Abdeckung), das Bonding erfolgte jedoch durch Kliniker mit unterschiedlicher Erfahrung. Die Gruppen 2, 3 und 4 unterscheiden sich im Design, wurden aber von demselben erfahrenen Kliniker geklebt

**Fig. 2:**
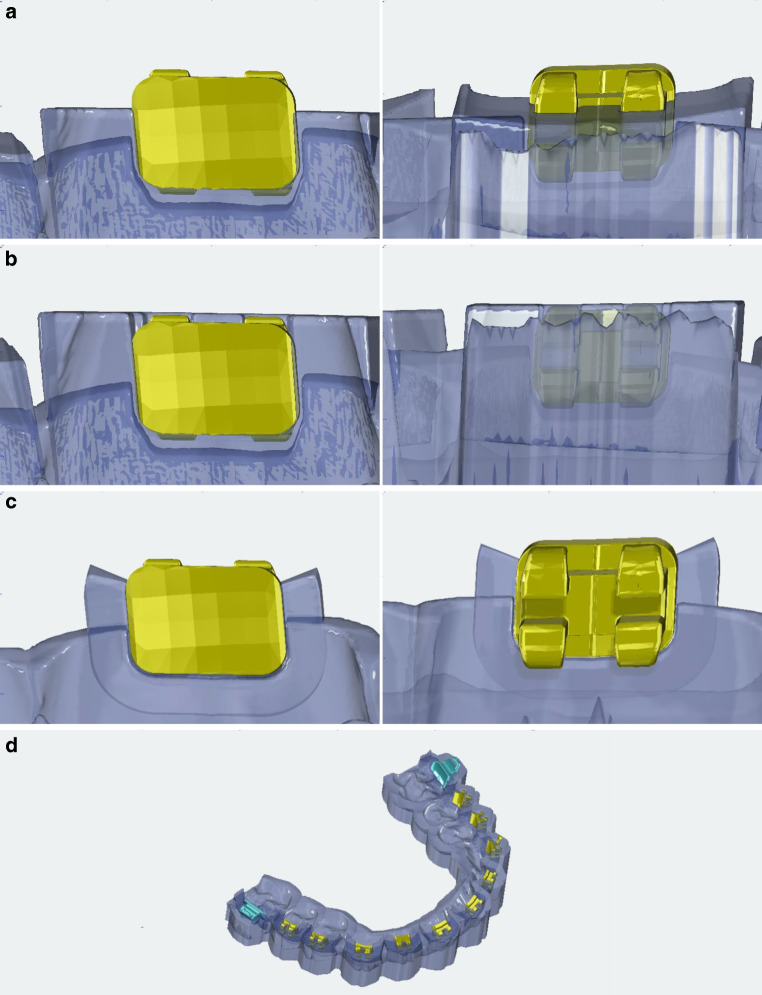
**a** Close-up view of design 1: palatal (*left*) and buccal (*right*). **b** Close-up view of design 2: palatal (*left*) and buccal (*right*). **c** Close-up view of design 3: palatal (*left*) and buccal (*right*). **d** View of the complete bonding tray (design 1) with 2.0 mm layer thickness and flat occlusal surface **a** Nahaufnahme von Design 1: palatinal (*links*) und bukkal (*rechts*). **b** Nahaufnahme von Design 2: palatinal (*links*) und bukkal (*rechts*). **c** Nahaufnahme von Design 3: palatinal (*links*) und bukkal (*rechts*). **d** Ansicht des kompletten Bondingtrays (Design 1) mit 2,0 mm Schichtstärke und flacher Okklusalfläche

Based on the virtual jaw model with brackets, three different types of bonding trays were designed in the software using the Bonding Trays 3D module (Fig. 1). Design 1 used a semi-open bracket coverage with 0.5 mm vertical slot overlap (groups 1, 2, Fig. 2a). Design 2 used a closed bracket coverage with 1.5 mm vertical slot overlap, which was investigated in a previous study (group 3, Fig. 2b; [12]). For design 3, an open design without bracket coverage was used (group 4, Fig. 2c).

Otherwise, the bonding trays were all designed according to the same parameters: distance to bracket 0.0 mm, rotation 100%, block-out 0.0 mm, thickness of 2.0 mm, and with a flat occlusal surface (Fig. 1d). Nesting and slicing were performed using Autodesk Netfabb software (version 2022; Autodesk Inc., San Rafael, CA, USA).

In all, 30 bonding trays of design 1 and 15 bonding trays of designs 2 and 3 were printed using a DLP 3D printer (SolFlex 350, W2P Engineering GmbH, Vienna, Austria) with a z-resolution of 50 µm. All samples were printed directly on the build platform of the printer. An experimental bonding tray material (Voco GmbH, Cuxhaven, Germany) was used for printing. Postprocessing was performed according to the manufacturer’s specifications.

### Bracket bonding procedure

The bonding trays were used to transfer and bond corresponding metal brackets on plaster duplicates of the digitized jaw model using composite (BrackFix, Voco GmbH, Cuxhaven, Germany). In groups 1, 2, and 3 the brackets were first fitted into the bonding trays before the trays were placed on the jaw models. In group 4, the bonding trays were first placed on the jaw model and the brackets were then positioned using an instrument and the seated tray as a positioning aid.

Groups 2, 3, and 4 were bonded by the same experienced orthodontic specialist with over 5 years of experience in indirect bonding (Fig. 1). Group 1 was bonded by a dentist who had no orthodontic experience but was trained in the use of the system through instruction and demonstration (Fig. 1). The number of bracket debondings during the procedure were recorded. These data were then utilized to determine the immediate bracket loss rate, calculated as the ratio of the number of bracket debondings to the number of brackets bonded.

### Accuracy assessment and statistical analysis

After the bonding procedure, the models with brackets were scanned with an industrial optical scanner ATOS 5 (GOM GmbH, Braunschweig, Germany) with a resolution of 12 µm. Accuracy of bracket placement was evaluated according to a published method using the software modules Register 3D and FA bonding 3D [12]: the scans were manually aligned with the reference data using anatomical reference points and subsequently matched to best-fit using an iterative closest point (ICP) algorithm (Register 3D module). Linear (mesiodistal, buccolingual, vertical) and angular (angulation, rotation, torque) deviations from the virtually planned positions were calculated using a software algorithm via coordinate transformation (FA bonding 3D module).

The measurement error associated with the utilized methodology was assessed in prior research using Bland–Altman statistics and a series of repeated measurements to determine the standard error of the mean (SEM) and confidence interval (CI) [19].

Clinically acceptable limit values were defined based on the American Board of Orthodontics (ABO) objective grading system for dental casts. Deviations of ≤ 0.5 mm and 2° were defined as clinically acceptable [14]. To account for the possibility of two adjacent brackets deviating in opposite directions, the limit of clinical acceptability in the present study was set at 0.25 mm and 1°. For the calculation of linear and angular offsets, as well for calculating the frequency of exceeding clinically acceptable limit values, all deviation values were set to their absolute values. This accounted for the possibility that some brackets have a positive offset of, for example, +1 mm, and others a negative offset of −1 mm, resulting in a higher distance of 2 mm comparing these two bracket positions.

Statistical analysis was carried out with SPSS Statistics (version 26, IBM, Armonk, NY, USA). Metric data were tested for normal distribution using the Shapiro–Wilk test. Significant differences in metric data were analyzed using the Kruskal–Wallis test and a post hoc Dunn–Bonferroni test. Nominal data were tested for significant differences using the Pearson χ^2^ test. A *P* value ≤ 0.05 was considered significant.

## Results

Table 1 shows the mean deviations of the bracket positions of the different tray designs between the digital design and the scanned bracket position after bonding. The mean deviations of the bracket positions of all bonding tray designs investigated were in a similar range (0.07–0.24 mm/0.42–1.90°). Significant differences were observed between the tree different tray designs (group 2–4) in the buccolingual (< 0.001), angulation (0.003), and rotational direction (< 0.001). Regarding the clinician experience groups (groups 1, 2) significant differences were observed in the mesiodistal (0.002), buccolingual (0.01), and torque direction (< 0.001).

**Table 1:** Mean (±SD) difference in mm and ° between the simulated bracket position and the postoperatively scanned bracket position of the different tray designs investigated. Significant differences between homogenous subgroups are indicated by superscript letters (α = 0.05) Mittlere (±SD) Differenz in mm und ° zwischen der simulierten Bracketposition und der postoperativ gescannten Bracketposition der unterschiedlichen untersuchten Tray-Designs. Signifikante Unterschiede zwischen homogenen Subgruppen sind durch hochgestellte Buchstaben gekennzeichnet (α = 0,05)

Dimension		Material						*p*-value					*p*-value
		Group 2		Group 3		Group 4			Group 1		Group 2		
		Mean	±SD	Mean	±SD	Mean	±SD		Mean	±SD	Mean	±SD	
*Linear error (mm)*	*Mesiodistal*	0.10	0.08	0.08	0.07	0.09	0.08	0.24	0.07	0.07	0.10	0.08	0.002
	*Vertical*	0.24	10.29	0.14	0.08	0.15	0.08	0.40	0.15	0.08	0.24	1.29	0.70
	*Buccolingual*	0.16^a^	0.08	0.21^a^	0.68	0.12^b^	0.09	< 0.001	0.14	0.07	0.16	0.08	0.01
*Angular error (°)*	*Angulation*	0.54^a^	0.42	0.64^ab^	0.48	0.84^b^	0.77	0.003	0.59	0.47	0.54	0.42	0.40
	*Torque*	1.25	0.87	1.09	0.74	1.25	1.05	0.30	1.90	1.02	1.25	0.87	< 0.001
	*Rotation*	0.88^a^	0.91	0.58^b^	0.42	1.00^a^	0.90	< 0.001	0.80	0.69	0.88	0.91	0.39

*SD *standard deviation, *Group 1* High bracket coverage (1.5 mm), inexperienced clinician; *Group 2* High bracket coverage (1.5 mm), experienced clinician; *Group 3* Low bracket coverage (0.5 mm), *Group 4* open

The mean deviations of the bracket positions of the different bonding tray designs investigated as well as of the different clinician experience subdivided by different tooth types are shown in Tables 2 and 3. Significant differences are observed mainly in the metric variables comparing the three different bonding tray designs (Table 2). There are no consistent differences with regard to the bonding tray designs within the tooth groups. Comparing professional experience, significant differences were observed in the mesiodistal and torque direction. Considering the torque values, group 1 showed significantly higher variations in each tooth group compared to group 2.

**Table 2:** Mean (±SD) difference in mm and ° between the simulated bracket position and the postoperative scanned bracket position of the tray designs investigated subdivided by different tooth types. Significant differences between homogenous subgroups are indicated by superscript letters (α = 0.05) Mittlere (±SD) Differenz in mm und ° zwischen der simulierten Bracketposition und der postoperativen gescannten Bracketposition der untersuchten Tray-Designs, differenziert nach den verschiedenen Zahntypen. Signifikante Unterschiede zwischen homogenen Subgruppen sind durch hochgestellte Buchstaben gekennzeichnet (α = 0,05)

Dimension		Mesiodistal				Vertical				Buccolingual				Angulation				Torque				Rotation			
Groups		2	3	4	*p*-value	2	3	4	*p*-value	2	3	4	*p*-value	2	3	4	*p*-value	2	3	4	*p*-value	2	3	4	*p*-value
Molar	Mean	0.20	0.19	0.18	0.60	0.12	0.17	0.12	0.06	0.07^a^	0.06^a^	0.13^b^	0.01	0.76^a^	0.79^a^	1.62^b^	< 0.001	1.66	1.61	1.65	0.87	1.16^ab^	0.57^a^	1.43^b^	0.01
	±SD	0.08	0.06	0.11		0.07	0.09	0.07		0.07	0.05	0.10		0.51	0.68	0.99		1.04	0.91	1.32		1.64	0.47	1.40	
Premolar	Mean	0.11	0.10	0.10	0.17	0.42^a^	0.11^a^	0.18^b^	< 0.001	0.17^a^	0.15^a^	0.07^b^	< 0.001	0.61	0.66	0.64	0.63	0.97	0.94	0.98	0.97	0.89^ab^	0.60^a^	1.13^b^	0.02
	±SD	0.05	0.05	0.07		2.20	0.07	0.08		0.07	0.05	0.07		0.44	0.43	0.57		0.71	0.70	0.75		0.66	0.33	0.87	
Canine	Mean	0.06	0.04	0.07	0.10	0.15	0.15	0.15	0.89	0.18^ab^	0.20^b^	0.15^a^	0.007	0.54	0.64	0.82	0.46	1.24	1.07	1.40	0.84	0.76	0.68	1.04	0.10
	±SD	0.05	0.04	0.05		0.07	0.07	0.07		0.11	0.07	0.08		0.41	0.54	0.70		0.76	0.54	1.33		0.80	0.52	0.75	
Incisor	Mean	0.04^a^	0.03^a^	0.06^b^	< 0.001	0.16	0.16	0.14	0.10	0.18^ab^	0.34^a^	0.14^b^	0.02	0.36^a^	0.56^b^	0.70^b^	0.004	1.34	0.98	1.26	0.12	0.79	0.53	0.65	0.09
	±SD	0.04	0.03	0.04		0.06	0.07	0.08		0.07	1.19	0.08		0.26	0.36	0.64		0.91	0.68	0.96		0.64	0.41	0.51	

*SD *standard deviation, *Group 2* High bracket coverage (1.5 mm), experienced clinician; *Group 3* Low bracket coverage (0.5 mm), *Group 4* open

**Table 3:** Mean (±SD) difference in mm and ° between the simulated bracket position and the postoperatively scanned bracket position of the same tray designs but different investigators subdivided by different tooth types (α = 0.05) Mittlere (±SD) Differenz in mm und ° zwischen der simulierten Bracketposition und der postoperativen gescannten Bracketposition der gleichen Tray-Designs, aber verschiedener Untersucher, differenziert nach verschiedenen Zahntypen (α = 0,05)

Dimension		Mesiodistal			Vertical			Buccolingual			Angulation			Torque			Rotation		
Groups		1	2	*p*-value	1	2	*p*-value	1	2	*p*-value	1	2	*p*-value	1	2	*p*-value	1	2	*p*-value
*Molar*	Mean	0.15	0.2	0.04	0.17	0.12	0.04	0.08	0.07	0.80	0.82	0.76	0.71	2.37	1.66	0.03	0.57	1.16	0.002
	±SD	0.09	0.08		0.09	0.07		0.07	0.07		0.60	0.51		1.32	1.04		0.80	1.64	
*Premolar*	Mean	0.07	0.11	< 0.001	0.11	0.42	0.06	0.12	0.17	< 0.001	0.54	0.61	0.47	1.59	0.97	< 0.001	0.69	0.89	0.11
	±SD	0.06	0.05		0.07	2.2		0.07	0.07		0.39	0.44		0.87	0.71		0.49	0.66	
*Canine*	Mean	0.06	0.06	0.79	0.15	0.15	0.79	0.15	0.18	0.19	0.55	0.54	0.90	2.08	1.24	< 0.001	1.06	0.76	0.75
	±SD	0.05	0.05		0.08	0.07		0.06	0.11		0.42	0.41		0.59	0.76		0.84	0.8	
*Incisor*	Mean	0.04	0.04	0.60	0.16	0.16	0.92	0.17	0.18	0.52	0.55	0.36	0.04	1.87	1.34	0.003	0.88	0.79	0.31
	±SD	0.04	0.04		0.07	0.06		0.06	0.07		0.49	0.26		1.07	0.91		0.68	0.64	

*SD *standard deviation, *Group 1* High bracket coverage (1.5 mm), inexperienced clinician; *Group 2* High bracket coverage (1.5 mm), experienced clinician

Table 3 summaries the prevalence of the clinically acceptable transfer errors of the different bonding tray designs (groups 2–4) and professional experience (groups 1 and 2). Most of the values of all groups were within the range of clinically acceptable limits regarding the linear values (93.0–97.8%). The angular values showed less variables within the range of clinically acceptable limits (19.3–88.8%). Especially in the torque direction, all groups showed the lowest agreement (19.3–57.1%). Group 4 exceeded the clinically acceptable limits significantly more frequently than the other groups in the angular and rotational direction. Group 1 exceeded the clinically acceptable limits significantly more frequently than group 2 in the vertical and torque direction.

Dividing the clinically acceptable limits into the tooth groups (Tables 4, 5, 6, 7, and 8), similar results can be observed. Group 4 is the only group that exceeded significantly more often the clinically relevant limits in all tooth groups compared to the other groups (groups 2 and 3). Group 1 exceeded significantly more often the clinically acceptable limits in the torque direction regarding all tooth groups compared to group 2.

**Table 4:** Prevalence of the clinically acceptable transfer errors of the different tray designs Prävalenz der klinisch akzeptablen Transferfehler der verschiedenen Tray-Designs

Dimension		Groups						
					*p*-value			*p*-value
		Group 2	Group 3	Group 4		Group 1	Group 2	
*Linear error (%)*	*Mesiodistal*	96.5	97.8	95.5	0.50	97.7	96.5	0.53
	*Vertical*	97.7	97.2	94.4	0.20	93.0	97.7	0.04
	*Buccolingual*	95.9	93.3	94.9	0.53	94.7	95.9	0.60
*Angular error (%)*	*Angulation*	87.8	83.1	68.9	< 0.001	87.8	87.8	0.98
	*Torque*	52.3	59.6	57.1	0.39	19.3	52.3	< 0.001
	*Rotation*	73.8	88.8	65.0	< 0.001	79.5	73.8	0.21

*Group 1* High bracket coverage (1.5 mm), inexperienced clinician; *Group 2* High bracket coverage (1.5 mm), experienced clinician; *Group 3* Low bracket coverage (0.5 mm), *Group 4* open

**Table 5:** Prevalence of the clinically acceptable transfer errors in the molar region of the tray designs investigated Prävalenz der klinisch akzeptablen Transferfehler im Molarenbereich bei den untersuchten Tray-Designs

Tooth		Molar						
					*p*-value			*p*-value
		Group 2	Group 3	Group 4		Group 1	Group 2	
*Linear error (mm)*	*Mesiodistal*	78.6	86.7	74.1	0.48	89.3	78.6	0.28
	*Vertical*	96.4	90	100	1.93	85.7	96.4	0.16
	*Buccolingual*	96.4	100	85.2	*0.05*	96.4	96.4	–
*Angular error (°)*	*Angulation*	71.4	76.6	33.3	*0.001*	71.4	71.4	–
	*Torque*	35.7	30	51.9	0.22	14.3	35.7	0.64
	*Rotation*	64.3	86.7	55.6	*0.03*	89.3	64.3	*0.03*

*Group 1* High bracket coverage (1.5 mm), inexperienced clinician; *Group 2* High bracket coverage (1.5 mm), experienced clinician; *Group 3* Low bracket coverage (0.5 mm), *Group 4* open

**Table 6:** Prevalence of the clinically acceptable transfer errors in the premolar region of the tray designs investigated Prävalenz der klinisch akzeptablen Transferfehler im Prämolarenbereich bei den untersuchten Tray-Designs

Tooth		Premolar						
					*p*-value			*p*-value
		Group 2	Group 3	Group 4		Group 1	Group 2	
*Linear error (mm)*	*Mesiodistal*	100	100	98.3	0.37	100	100	–
	*Vertical*	98.3	100	90	*0.01*	96.3	98.3	0.51
	*Buccolingual*	96.6	100	100	0.13	98.1	96.6	0.61
*Angular error (°)*	*Angulation*	86.4	83.3	83.3	0.87	94.4	86.4	0.15
	*Torque*	62.7	71.7	66.7	0.58	25.9	62.7	*<* *0.001*
	*Rotation*	71.2	91.7	58.3	*<* *0.001*	87	71.2	*0.04*

*Group 1* High bracket coverage (1.5 mm), inexperienced clinician; *Group 2* High bracket coverage (1.5 mm), experienced clinician; *Group 3* Low bracket coverage (0.5 mm), *Group 4* open

**Table 7:** Prevalence of the clinically acceptable transfer errors in the canine region of the tray designs investigated Prävalenz der klinisch akzeptablen Transferfehler im Eckzahnbereich bei den untersuchten Tray-Designs

Tooth		Canine						
					*p*-value			*p*-value
		Group 2	Group 3	Group 4		Group 1	Group 2	
*Linear error (mm)*	*Mesiodistal*	100	100	100	–	96.7	100	0.32
	*Vertical*	93.1	96.7	96.7	0.75	93.3	93.1	0.97
	*Buccolingual*	93.1	83.3	93.3	0.34	93.3	93.1	0.97
*Angular error (°)*	*Angulation*	86.2	80	60	*0.05*	86.7	86.2	0.96
	*Torque*	51.7	56.7	53.3	0.93	3.3	51.7	*<* *0.001*
	*Rotation*	75.9	80	60	0.92	63.3	75.9	0.30

*Group 1* High bracket coverage (1.5 mm), inexperienced clinician; *Group 2* High bracket coverage (1.5 mm), experienced clinician; *Group 3* Low bracket coverage (0.5 mm), *Group 4* open

**Table 8:** Prevalence of the clinically acceptable transfer errors in the incisor region of the tray designs investigated Prävalenz der klinisch akzeptablen Transferfehler im Inzisivenbereich bei den untersuchten Tray-Designs

Tooth		Incisor						
					*p*-value			*p*-value
		Group 2	Group 3	Group 4		Group 1	Group 2	
*Linear error (mm)*	*Mesiodistal*	100	100	100	–	100	100	–
	*Vertical*	100	98.3	95	0.19	93.2	100	*0.05*
	*Buccolingual*	96.4	87.9	95	0.16	91.5	96.4	0.27
*Angular error (°)*	*Angulation*	98.2	87.9	75	*0.001*	89.8	98.2	0.06
	*Torque*	50	63.8	51.7	0.27	23.7	50.0	*0.003*
	*Rotation*	80.4	91.4	78.3	0.13	76.3	80.4	0.60

*Group 1* High bracket coverage (1.5 mm), inexperienced clinician; *Group 2* High bracket coverage (1.5 mm), experienced clinician; *Group 3* Low bracket coverage (0.5 mm), *Group 4* open

Table 9 summarizes the prevalence of the immediate bracket losses. No significant differences were observed between the different bonding tray designs, neither in the overall analysis nor in the subdivision into the tooth groups. In terms of professional experience (groups 1–2), group 1 demonstrated a significantly lower bracket loss rate for premolars compared to group 2.

**Table 9:** Immediate bracket loss rate (%) regarding tray designs, clinician experience and different tooth types Rate der sofortigen Bracketverluste (%) in Abhängigkeit von Tray-Design, klinischer Erfahrung und verschiedenen Zahntypen

	Bracket loss rate in %			*p*-value	Bracket loss rate in %		*p*-value
	Group 2	Group 3	Group 4		Group 1	Group 2	
*Total*	4.4	1.1	1.7	0.07	5.0	4.4	0.74
*Molar*	6.7	0	10.0	0.23	6.7	6.7	–
*Premolar*	1.7	0	0	0.37	10.0	1.7	*0.05*
*Canine*	3.3	0	0	0.36	0	3.3	0.31
*Incisor*	6.7	3.3	0	0.13	1.7	6.7	0.17

*Group 1* High bracket coverage (1.5 mm), inexperienced clinician; *Group 2* High bracket coverage (1.5 mm), experienced clinician; *Group 3* Low bracket coverage (0.5 mm), *Group 4* open

## Discussion

All bonding tray designs investigated resulted in accurate bracket placement. The mean deviations of the bracket positions of all materials investigated were in a similar range. However, the angular variables showed a higher magnitude of error than the linear variables (0.04–0.21 mm/0.20–1.75°). Similar findings were observed regarding clinically acceptable limits. Most of the values were within the range of clinically acceptable limits regarding the linear values (93.0–97.8%). The angular values showed fewer variables within the range of clinically acceptable limits (19.3–88.8%). Especially in the torque direction, all groups showed the lowest precision (19.3–57.1%).

Those results are in line with previous literature investigating 3D-printed bonding trays for indirect bonding [4, 12, 14, 17, 21]. Bachour et al. evaluated the transfer accuracy of 3D-printed indirect bonding trays using a fully digital workflow. The authors observed the highest transfer accuracy for mesiodistal and buccolingual bracket placement (both 100% within the clinically acceptable limits) and the lowest for torque (46.0% within the clinically acceptable limits). Niu et al. observed that only 57% of the cases demonstrated clinically acceptable bracket positioning for tip, 51% for torque, and 85% for rotation. Similarly, Kim et al. reported a high transfer accuracy within the clinically acceptable limits (93–100%) in the linear dimensions, but the transfer accuracy in the angular dimensions was low (27–57%).

Bachour et al. concluded that the intraoral scanner may not be able to accurately reproduce angular measurements. However, in this study, a high-resolution industrial scanner (ATOS 5) was used to analyze the bracket position, whereby this influencing factor was presumably minimized. Niu et al. attributed the high transfer accuracy in linear areas and the low transfer accuracy in angular areas to the material properties of the bonding trays. Harder tray materials may exhibit better angular transfer accuracy. However, this can result in higher friction between the brackets and the bonding tray, potentially leading to a higher rate of bracket debonding during tray removal.

The mean deviations of the bracket positions of the different bonding tray designs considered in the individual tooth groups showed that in the mesiodistal dimension, molars exhibited the highest inaccuracy within all bonding trays investigated (0.18–0.20 mm). A common explanation for higher transfer inaccuracies of molars is the difficulty in maintaining the same pressure on the entire tray during the transfer, especially in the hard-to-reach posterior regions [7, 11, 12]. However, in the other linear and angular dimensions, no tooth groups consistently showed better or worse values compared to each other. Therefore, except for the mesiodistal dimension, no influence on the bracket placement accuracy seems to occur depending on the tooth group.

Comparing the bonding trays to each other, group 4 achieved significantly better results in the buccolingual dimension for almost all tooth groups. This could be due to the open design, which allowed a better bracket adaptation to the tooth surface, resulting in improved outcomes in the buccolingual dimension. However, group 4 showed a significantly more frequent exceeding of the clinically relevant limits compared to the other groups. The open design of group 4 seems to allow good buccolingual control, however, is accompanied by too large a leeway for the other dimension resulting in a frequent exceeding of the clinically relevant limits.

Considering the influence of professional experience, group 1 exhibited significantly worse results in the torque dimension and significantly better results in the buccolingual and mesiodistal dimensions compared to group 2. However, these linear differences were within the range of 30 µm and thus considered clinically irrelevant. However, regarding the total prevalence of the clinically acceptable transfer errors, group 1 performed significantly worse exceeding the clinically acceptable limits in the vertical and torque dimension compared to group 2 (Table 3). To date, there is limited literature regarding the influence of the investigator on the accuracy of bracket placement using the indirect bonding technique [3]. Armstrong et al., investigated the accuracy of bracket placement by orthodontists and inexperienced dental students and concluded that an accurate direct bonding of orthodontic brackets to teeth does not appear to be related to clinical experience or specialist training. However, based on this study, it appears that clinical experience seems to have an influence on the exceedance of clinically relevant limits, as group 1 exceeded the clinically acceptable limits significantly more frequently than group 2 in the vertical and torque directions.

The immediate bracket loss rate was between 1.7 and 6.7% for all bonding tray designs investigated. No significant differences could be observed between the different bonding tray designs, neither in the overall analysis nor if considered in the individual tooth groups. These findings are in line with the current literature postulating a loss rate up to 11% [4, 5, 12, 17, 18]. Czolgosz et al. showed in a randomized controlled trial an immediate bracket loss rate of 5.1% compared to 0% bracket loss rate using the direct bonding method. They concluded that the indirect bonding method may be used with a too short light-curing time due to the printed bonding trays [8]. In the context of this study, the bracket-loss rate could have been related with a reduced bond strength as a result of bonding the brackets with composite to plaster surfaces.

In terms of professional experience (groups 1 and 2), group 1 demonstrated a significantly lower bracket loss rate for premolars compared to group 2. However, regarding the total analysis of bracket loss rate no significant differences could be observed between groups 1 und 2. Thus, a slightly higher bracket loss rate by inexperienced orthodontists can be assumed.

## Conclusion

All bonding tray designs investigated resulted in an accurate bracket placement. However, the open design (group 4) appeared to be inferior to the other bonding trays in terms of exceeding clinically relevant limits. The experience of the orthodontist has an impact on the accuracy of bracket placement and also on the immediate bracket loss rate. Therefore, selection of the correct bonding tray design as well as the clinical experience and expertise in indirect bonding procedures is important for achieving optimal bracket placement accuracy.

## Data Availability

The datasets used are available from the corresponding author on request.
